# Quantitative determination of intracellular miRNA content using dual gold and iron nanoreporters and single particle ICP-ToF–MS

**DOI:** 10.1007/s00604-025-07236-4

**Published:** 2025-06-02

**Authors:** Sara González Morales, Elena Añón Álvarez, David Clases, Mario Corte-Rodriguez, Maria Montes-Bayón

**Affiliations:** 1 Department of Physical and Analytical Chemistry, Faculty of Chemistry, University of Oviedo, Julián Clavería 8, Oviedo, 33006 Spain; 2 Health Research Institute of the Principality of Asturias (ISPA), Av. Hospital Universitario s/n, Oviedo, 33011 Spain; 3 Insitute for Chemistry, Karl-Franzens-Universität Graz, Graz, 8010 Austria

**Keywords:** miRNA, Cell lines, Au/Fe simultaneous detection, Time-of-flight ICP-MS

## Abstract

MicroRNAs (miRNAs) are short single-stranded RNA sequences that play an important role in the initiation and progression of cancer. Therefore, the present work tries to establish an analytical platform for the quantitative determination of miRNA in cancer cell models without enzymatic amplification reactions. The developed assay is based on a sandwich double-hybridization reaction using a capture oligonucleotide conjugated to magnetic iron oxide microparticles and detecting oligonucleotide conjugated to a 40-nm gold nanoparticle, both particles coated with streptavidin. The optimization of the double-hybridization assay is conducted using inductively coupled plasma in single particle mode with a time-of-flight analyzer (SP-ICP-ToF–MS) for double detection of Au and Fe within the same event. The developed strategy was directly applied to the quantification of miR-16-5p in cell lysates without amplification reactions. For this aim, the cancer cell line of melanoma (A375) was studied, and two sample preparation strategies have been evaluated. Sequence capturing in extracted RNA provided best results allowing the determination at about 200 pM of miR-16-5p (for 2 × 10^6^ cells). This strategy represents one of the few alternatives to obtain absolute quantification of miRNA in biological samples to permit the direct comparison among cell lines without amplification or transformation reactions of the original sequence.

**Graphical Abstract:**

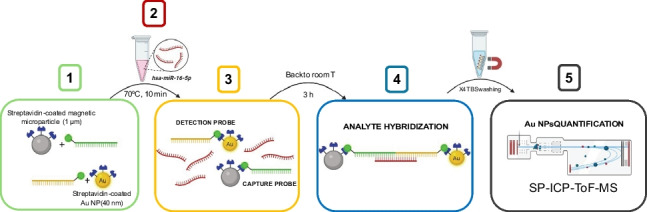

**Supplementary Information:**

The online version contains supplementary material available at 10.1007/s00604-025-07236-4.

## Introduction

MicroRNAs (miRNAs) are small-sized transcripts that regulate expressions of genes at post-transcriptional level through specific targeting of mRNAs. Typically, miRNAs appear with lengths between 21 and 25 nucleotides. They are the product of a multi-step synthesis that involves nuclear and cytoplasmic proteins [1]. Some of these sequences are known to be involved in the carcinogenic process, since they regulate the expressions of several oncogenes and tumor suppressor genes as well as the activity of cancer-associated pathways [2, 3]. In particular, miR-16-5p is an example of a miRNA that plays important roles in the development of diverse malignancies including osteosarcoma, neuroblastoma, lung cancer, breast cancer, bladder cancer, gastrointestinal cancers, hepatocellular carcinoma, and gastrointestinal cancers [4, 5]. Specific studies in osteosarcoma cells have shown that the upregulation of miR-16-5p suppressed proliferation, migratory potential, and invasive features of this malignancy and increased the cytotoxic effects of cisplatin on these cells [6]. In breast cancer cells, reduced expression of miR-16-5p has been linked to enhanced cell migration and proliferation, accelerated cell cycle progression, and decreased rates of apoptosis [7]. Therefore, miRNAs have great potential as diagnostic or prognostic biomarkers but also as novel drugs or therapeutic targets. However, these promising clinical applications of miRNAs require methods to quantify expression levels accurately and reproducibly [8].

In general, quantification of miRNAs is possible by using hybridization, amplification, sequencing, and enzyme-based methods [9, 10]. Real-time quantitative polymerase chain reaction (RT-qPCR) is most commonly used for targeted quantification of miRNAs as a low-to-medium throughput method. However, variations in primer design and inconsistent data analysis and normalization can negatively affect the reproducibility of RT-qPCR [11]. Nonetheless, RT-qPCR is currently applied as the gold standard method for miRNA quantification and as verification for other techniques like microarrays or next-generation sequencing. The use of metallic nanoparticles for miRNA detection represents also a growing trend. Spectroscopic detection of miRNA based on colorimetric methods [12, 13] or light scattering [14] of gold nanoparticles have been reported. Recently, alternative strategies using such nanostructures as labels for elemental analysis using plasma mass spectrometry as detection method have been also implemented for miRNA or short RNA/DNA sequences in biological samples [15–17]. Previously, our group developed one of these platforms to address the presence of circulating miR-16-5p in blood serum by using SP-ICP-MS with gold nanoparticles as elemental labels [18]. That work presented the added value of mass spectrometric detection and was validated against RT-qPCR for the same set of samples with satisfactory quantitative results without amplification or transformation of the sought analyte.

In this study, we attempted the analysis of miR-16-5p in cell cultures, which represents a more complex and demanding system. For this purpose, we have used a modified double hybrid sandwich assay and employed a new generation of ICP-MS equipped with a time-of-flight (ToF) mass analyzer. Such configuration permits the simultaneous monitoring of Au and Fe in the single event of the hybrid and avoids its disassembling, increasing the selectivity of the measurement. An instrument with a ToF analyzer provides new opportunities for single particle-based applications as they do not only have very fast spectra acquisition at 10 kHz and beyond but also record virtually any isotope across a mass range of 7–275 amu [19–21].

One important advantage of this platform is that on each individual experiment (calibration and sample), the analyte is recognized and captured using the same set of gold nanoparticles and magnetic Fe microparticles. As such, the stoichiometry of the number of probes per particle (nano or micro) does not need to be known. Similarly, the transport efficiency of gold nanoparticles to the ICP-MS does not need to be considered either, as every data point of the calibration curve and the sample will be affected by this efficiency in the same way [22]. However, a challenge is to increase the sensitivity of the assay aiming, in an ideal case, to “single molecule” detection by minimizing the number of probes per gold nanoparticle. Therefore, the optimization of the labelling of the Au-NPs with the probe is here carefully conducted using miR-16-5p as a model molecule. Under the optimum conditions, the performance of the double hybrid sandwich assay using SP-ICP-To-MS is then applied for the determination of miR-16-5p in cell lysates of melanoma cell line using optimized sample preparation strategies and increased selectivity by using the simultaneous double detection of Fe and Au. This work aims to illustrate the possibilities of ICP-MS based bioassays for absolute miRNA quantification that can be extended to cells of different origins allowing comparative results using absolute concentration units.

## Experimental section

### Instrumentation

For the characterization of the assay via SP-ICP-ToF–MS, a Vitesse ICP-ToF–MS system by Nu Instruments (Wrexham, UK) was operated in single particle mode recording, binning (3 spectra) and saving mass spectra from 20 to 240 amu at 12.85 kHz (corresponding to a spectra saving interval of ~ 80 µs), while blanking the ranges 24.5–30.5 and 38–47 amu to avoid signal saturation at the detector. Data acquisition was performed by Nu Codaq Vitesse software (Nu Instruments) (version 1.5.8267.1), and SP ICP-ToF–MS raw data was directly processed by a modified version of SPCal (ver. 1.1.2), developed by Lockwood et al. [23, 24] and adapted for ICP-ToF–MS data structure. Decision limits were determined using compound Poisson sampling of a lognormal approximation of the signal ion distribution. The plasma was operated at 1.35 kW and the segmented reaction cell was operated with helium (12 mL min^−1^) and hydrogen (8 mL min^−1^) as cell gases. Instrumental parameters such as nebulizer Ar flow and torch position were optimized daily to obtain the best sensitivity.

For SP analysis, the ICP-ToF–MS system was equipped with a concentric nebulizer (Glass Expansion, Weilburg, Germany) and a cyclonic spray chamber. The nebulizer flow rate was tuned to provide the highest sensitivities while maintaining a CeO/Ce ratio below 10%. The aerosol’s transport efficiency was determined by analyzing a diluted 80 nm Au NP standard (nanoComposix, California, USA) and ionic standards of known concentration, using an automated approach via the SP data processing platform SPCal. Dilutions were performed in tubes made of polypropylene. Each sample, blank and calibration standard was recorded for 120 s. The ICP-ToF–MS conditions are shown in Table 1. A NanoDrop spectrophotometer (Thermo Fisher Scientific) was used for the spectrophotometric determination of nucleic acid concentrations.

**Table 1:** Operating conditions of the ICP-Tof–MS used in the study

REACTION CELL-OVERVIEW		
Parameters	**Au 50 nm**	**Au 100 nm**
RF set (V)	2.0	2.0
RF power (W)	1350	1350
Peripump speed (rpm)	50	50
Neb flow (mL min^−1^)	1150	1150
Sample flow rate (mL min^−1^)	0.2	0.2
Entrance aperture (V)	** − **45.0	** − **45.0
Cell entrance (V)	** − **5.5	** − **3.0
Entrance offset (V)	6.0	5.0
Exit offset (V)	** − **12.0	** − **11.0
Cell exit (V)	** − **8.5	** − **10.0
Exit aperture (V)	** − **35.0	** − **30.0
Helium cell gas flow (mL min^−1^)	13.0	12.0
Hydrogen cell gas flow (mL min^−1^)	9.0	8.0
LOD (nm)	24.6	27.5

### Materials and reagents

All solutions were prepared in ultrapure water using a PURELAB flex 3 apparatus from Elga Veolia (High Wycombe, UK). DEPC-treated water (Ambion and Life Technologies) was used for solutions needed to preserve miRNA samples. Low protein binding microcentrifuge tubes from ThermoFisher Scientific were used to minimize the level of RNA-unspecific binding to plastic surfaces.

Different gold nanoparticles have been used in this study: (1) the gold nanoparticles used for conjugation with the probe were 40-nm gold nanospheres coated with streptavidin OD10 from CD Bioparticles (New York, USA); (2) 80-nm Au NP standard from NanoComposix (San Diego, USA), characterized regarding size distribution, optical properties, surface potential, and hydrodynamic radius by the manufacturer, to address transport efficiency in SP ICP-ToF–MS. Magnetic microparticles were SpeedBead Magnetic Streptavidin-coated particles of 1-µm size from Nano Composix.

Ionic gold 1000 ppm standard NIST3121 from Merck was used for calibration. All ionic solutions were diluted in 2% nitric acid prepared from 65% HNO_3_ from Acros Organics (Geel, Belgium), previously purified by sub-boiling distillation. Argon gas for the operation of ICP-MS with 99.999% purity was supplied by Air Liquide (Paris, France). Tris buffer saline (TBS) was purchased as a soluble tablet from Sigma-Aldrich. Phosphate buffer and aging buffer were prepared in-house using inorganic salts from Merck. Tween 20 was obtained from Sigma-Aldrich. TRIzol reagent from Invitrogen was used for the extraction of RNA from cells, in addition to chloroform, isopropanol, and 75% ethanol in RNase-free water, all of them from Sigma-Aldrich.

Elemental standards at 1000 μg L^−1^ for ICP-MS (Single-Element ICP-Standard-Solution Roti®Star), diluted to working conditions using ultra-pure water (18.2 MΩ cm; Merck Millipore, Bedford, USA), were used.

### Methodology

**Scheme 1:**
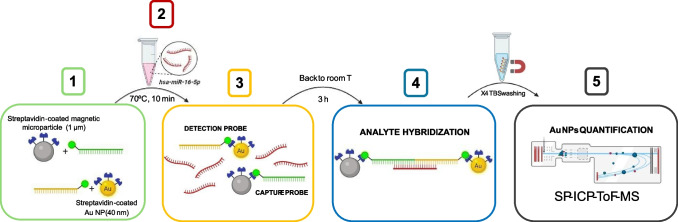
General workflow of the hybridization assay

The nanoparticle-based miRNA determination assay consists of the following steps (see Scheme 1):

1. Preparation of detection and capture probes.
2. Incubation of the probes with the sample at 70 °C for 10 min.
3. Slow cooling (3 h) of the mixture down to room temperature.
4. Washing the sample four times with Tris buffer saline (TBS), using a magnet to retain the sandwich formed by the analyte and the detection and capture probes.
5. Adequate dilution of the washed sandwich and determination of nanoparticle concentration by SP-ICP-ToF–MS.

#### Oligonucleotides

DNA oligonucleotides were custom-made by Invitrogen (Massachusetts, USA) as lyophilized powder. The different sequences used in this study are shown in Table 2: the target RNA miR-16-5p sequence (1), a biotinylated DNA oligo that is complementary to the 3′ end of the target miRNA (capture probe) (2), and the also biotinylated half-complementary sequence to the 5′ end of the target miRNA (detection probe) (3). In order to avoid degradation problems of the ARN during the optimization, all previous steps were performed using the equivalent DNA sequence for miR-16-5p (1*). This is possible because the hybridization of RNA with DNA also has a high affinity. For the same reason, both detection and capture probes were used as DNA oligos, even when real miRNA samples were used.

**Table 2:** DNA sequences used in the work of the target analyte, capture and detection probes

NAME	SEQUENCE
(1) miR-16-5p (target)	5′-UAG CAG CAC GUA AAU AUU GGC G-3′
(1)* miR-16-5p (surrogate DNA target)	5′-TAG CAG CAC GTA AAT ATT GGC G-3′
(2) Capture oligo (biotinylated)	5′-T TTA TAA CCG CAA (AAA)_7_ -BIOT-3′
(3) Detection oligo (biotinylated)	5′-BIOT-(AAA)_8_ ATC GTC GTG CA-3′

The capture and detection sequences were extended with homopolymeric stretches of adenine triplets—seven (21 adenines) for the capture probe and eight (24 adenines) for the detection strand. This modification was introduced to distance the hybridizing region from the labeling moiety, thereby minimizing steric hindrance. Full sequence details are provided in Table 2.

#### Preparation of the detection probe

The detection probe consists on the conjugation of the detection biotinylated oligo with the 40-nm streptavidin-coated gold nanoparticles. For this, 250 μL of the gold nanoparticles was centrifuged at 10,000 rpm for 5 min. The supernatant was removed and the nanoparticles were re-suspended in TBS buffer containing 0.01% Tween 20 for a higher stability. This suspension was mixed with 16.7 μL of the detection biotinylated oligo and incubated for 30 min at room temperature. The freshly assembled detection probe was washed by centrifugation for 5 min at 10,000 rpm and re-suspended in 500 μL of TBS. The number of washing steps was optimized to four.

#### Preparation of the capture probe

To prepare the capture probe, the biotinylated capture oligonucleotide was linked to streptavidin-coated magnetic microparticles. A volume of 4 μL of magnetic beads was subjected to three washing cycles using a magnetic separator and a buffer composed of 2 M NaCl, 1 mM EDTA, and 10 mM Tris (pH 7.5, prepared with ultrapure water). Subsequently, 92 pmol of the oligonucleotide was mixed with the microparticles and allowed to react for 20 min at ambient temperature. Unbound oligos were removed through two additional washes, using a magnet to retain the functionalized microparticles.

#### Hybridization assay

The probes were incubated with 100 µL of sample (cell lysate) at 70 °C for 10 min. This step allows to denature any hybridization or secondary structures of probes or analytes. Although typical denaturalization steps for DNA go up to 100 °C, in this case, the temperature must be kept below 80 °C to preserve the structure of biotin. After this time, the mixture is slowly cooled down during 3 h down to room temperature. A slow cooling is crucial to achieve the hybridization of the nucleic acid sequences by base complementarity, ensuring a highly selective interaction. Once the mixture is cool, the excess of reagents is washed at least four times with TBS. Since the sandwich will be retained using a magnet thanks to the magnetic microparticles, all other components, specially non-bound gold nanoparticles, will be washed from the solution.

#### Single Particle ICP-ToF–MS measurement (data acquisition and processing)

SP-ICP-ToF–MS enables the rapid acquisition of full mass spectra, a critical requirement for fast non-target screenings of particulate elements [20]. However, its potential is partially limited by the generation of large data files that can reach several gigabytes per sample. To address this, we used SPCal software [24], which incorporates various tools for the processing of large SP-ICP-ToF–MS data. This involved multiple and iterative data processing steps, contributing to find SP signals, fit and smooth raw data, accumulate data points of individual SP signals, perform calibrations (e.g., mass, size, composition), calculate key parameters (e.g., limits of detection (LOD), transport efficiency, ionic response), and visualize data (histograms, charts, etc.).

The analysis and of the 40-nm gold nanoparticles and the validation of the assay were conducted using SP-ICP-ToF–MS. Thanks to the time-of-flight mass analyzer and the optimized ion optics, it was possible to detect multiple elements simultaneously from the ionization of individual nanoparticles, using short integration times and substantial sample dilution. The transient signals generated as single nanoparticles entered the plasma were quantified and translated into gold mass using an external calibration curve based on ionic gold. This conversion accounted for the transport efficiency of the ionic standards, which was determined daily using 80-nm gold nanoparticles and ionic solutions of known concentrations. With the gold mass per particle calculated and assuming spherical shape and pure gold composition, both the volume and corresponding particle diameters were derived.

The result of the hybridization assay (the sandwich containing the analyte, the capture and the detection probe) after the washing steps was diluted in water and introduced into the ICP-ToF–MS, and the signal of the whole mass range was acquired for 120 s. After the measurement, the signal was processed using the software SPCal in order to separate the events from the background. For this aim, SPCal followed an iterative algorithm based on Gaussian statistics to establish a threshold above which a signal is considered an event based on Au. This first data filtration provided the number of gold-containing events. The same software was then used to count only those events containing both gold and iron to obtain the number of Au + Fe events. Only the latter corresponds to the double positively detected target miRNA, identified by both the capture and the detection probe.

#### Cell cultures

The A375 cell line was cultured in DMEM medium supplemented with 10% heat-inactivated fetal bovine serum and 5 mg·L^−1^ Plasmocin. Cells were grown in T25 flasks within a humidified incubator maintained at 37 °C and 5% CO_2_. The culture medium was refreshed every 2–3 days. When cells reached 80–90% confluency, they are subcultured. This involves removing the spent medium, washing the flask with PBS, and treating with 1 mL of 0.25% trypsin–EDTA for approximately 5 min to detach the cells from the flask surface. Following trypsinization, 5 mL of fresh DMEM was added to neutralize the trypsin and cells were pelleted by centrifugation at 400 g for 5 min. A 1:5 dilution of the cell suspension was then seeded into a new T25 flask. This process was repeated as needed to obtain the desired number of cells for the experiments.

For every assay, the collected cells were counted using a Neubauer chamber. After trypsinization and pelleting by centrifugation, cells were appropriately diluted and 10 µL of this suspension was loaded into each chamber of the hemocytometer and observed under a microscope, counting viable cells within the four corner squares and the central square, consistently applying the same criteria for cells on the borders. Finally, cell concentration was calculated by averaging the number of cells per square, applying the corresponding dilution factor.

#### Cell lysis

A cell lysis methodology was employed, based on freeze/thaw cycles in ultrapure water to disrupt cell walls through osmosis and cyclic crystallization/melting. For this purpose, the treated cell pellet was resuspended in 1 mL of ultrapure water. Five freezing cycles in liquid nitrogen and thaw cycles in a 50 °C water bath were performed on the cell suspension. Subsequently, the resulting solution was centrifuged for 10 min at 5000 g, yielding a precipitate of cell debris and organelles, and a supernatant containing the analyte with other ionic compounds and proteins present in the cytosol. This extract was used as such to attempt the capturing of the analyte.

#### Sample pre-treatment for RNA isolation

Total cytosolic RNA was extracted from cultured cells using TRIzol reagent following standard procedures. Briefly, cells were harvested from culture flasks by trypsinization and pelleted by centrifugation at 400 g for 5 min. The cell pellet was then lysed in 1 mL of TRIzol reagent per 1–5 × 10⁶ cells, homogenized and incubated at room temperature for 5 min to dissociate nucleoprotein complexes. To separate phases, 0.2 mL of chloroform per 1 mL of TRIzol was added, followed by vigorous shaking and a 2–3-min incubation at room temperature. After centrifugation at 12,000 × *g* for 15 min at 4 °C, the aqueous phase containing RNA was carefully transferred to a new tube, and RNA was precipitated by adding 0.5 mL of isopropanol per 1 mL of TRIzol. The sample was incubated at room temperature for 10 min, followed by centrifugation at 12,000 × *g* for 10 min at 4 °C. The RNA pellet was washed with 75% ethanol, air-dried, and resuspended in 100 μL RNase-free water. RNA concentration and purity were assessed by spectrophotometry at 260/280 and 260/230 nm, respectively.

## Results and discussion

### Influence of the size of Au NPs and magnetic microparticles on the assay performance

The scheme of the sandwich double hybridation assay is shown in Figure S1. It is worth mentioning that the initial assay (optimized using a ICP-TQ-MS) included the use of thiolated Au-NPs with a nominal size of 25 nm [18]. However, the size detection limit obtained with the ICP-ToF–MS for gold nanoparticles, which turned out to be around 25 nm, did not allow their use. Therefore, an initial modification of the assay was carried out to use the new Au-NPs of larger diameter and containing streptavidin instead of thiolate groups. In this case, the recognition probe had to be equally modified to a sequence containing biotin instead of thiol groups at the end for conjugation with the NPs (Table 2). Therefore, the new system needed to be first characterized.

In addition, when using SP-ICP-ToF–MS, the two elemental labels of the final product after the analyte was captured (Au and Fe) can be simultaneously monitored while this cannot be done when using SP-ICP-MS (where the disassembling of the sandwich assay before analysis was required). Such possibility allowed a more selective study of the blank contribution, since detected particles containing only gold or iron would not be forming the sandwich including the analyte. Thus, different sized Au-NPs were tested (40 and 60 nm), both coated with streptavidin, to be labelled with the biotinylated corresponding oligonucleotide to obtain the detection probe. The suitability of the instrument for the accurate measurements of both sized particles was tested using the conditions previously described for SP-ICP-ToF–MS (see Table 1). The size histograms (see Figure S2) showed a mean size of 40.7 ± 5.4 nm for the 40 nm Au NPs and 59 ± 13 nm for the 60 nm, which fitted well to the values provided by the manufacturers. Therefore, both particle suspensions were used as detection probes.

**Fig. 1:**
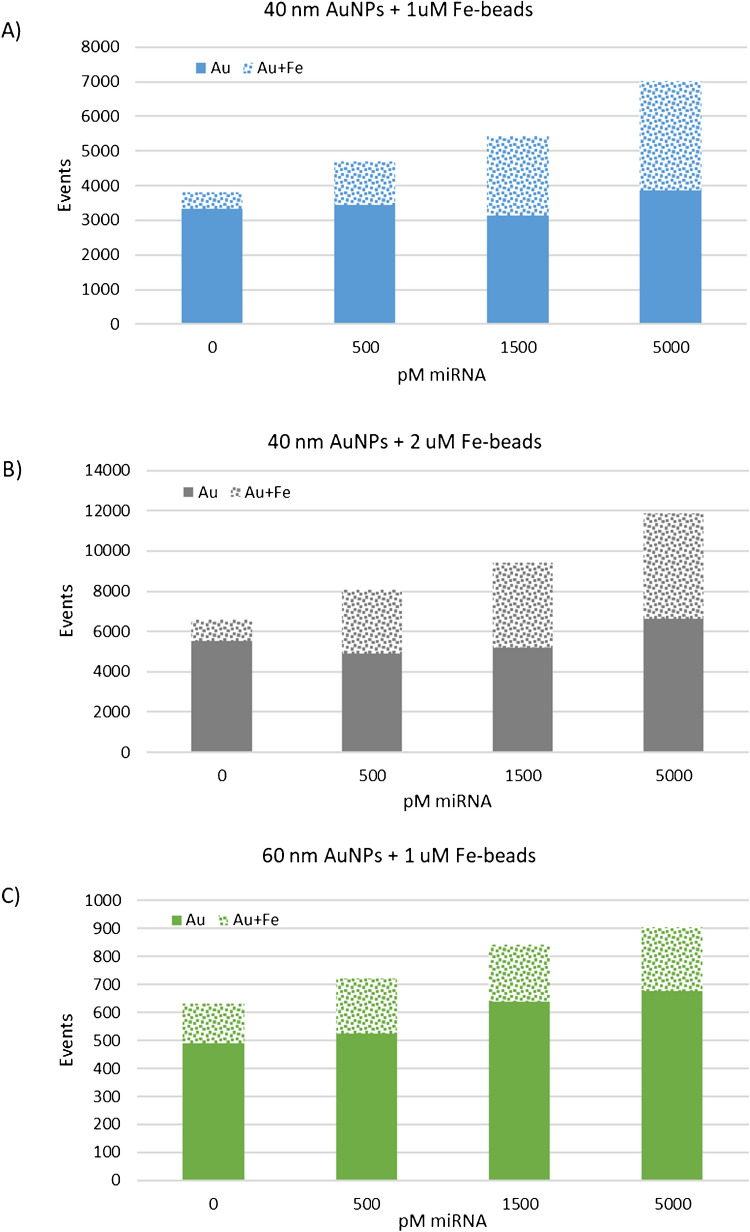
Number of events for the different combinations of detection and capture probes under evaluation represented as the number of detected Au events (solid bar) and events containing both Au and Fe (dotted bar, Au + Fe events) versus increasing concentrations of the miRNA under study. The combinations correspond to **A** 40 nm AuNPs + 1uM beads, **B** 40 nm AuNPs + 2uM beads, and **C** 60 nm Au NPs + 1uM beads

Similarly, two different sized streptavidin-coated magnetic beads (1 and 2 µm, respectively) were tested for conjugation to the biotinylated oligonucleotide to produce the capture probe. Figure 1A, B and C show the obtained results for the different combinations of detection and capture probes under evaluation represented as the number of detected Au events (solid bar) and events containing both Au and Fe (dotted bar, Au + Fe events) versus increasing concentrations of the target miRNA, not hybridized with the analyte or the capture probe. Gold events that were coincident with iron could be ascribed to be corresponding to the sandwich formed by the detection probe, the miRNA, and the capture probe.

Only this combined Au + Fe events should be counted in the quantification of the analyte. In this regard, a small fraction of the gold events did also contain Fe (about 450 events in Fig. 1A and up to 6000 events in Fig. 1B) even in the absence of the analyte, ascribed to unspecific interactions between the two probes. The lowest blank levels were observed when applying 60-nm gold probes and 1 µM Fe beads but no increasing response was observed (regarding Au + Fe events) upon varying the miRNA concentration. Finally, the combination of 40-nm gold probes and 1 µM Fe beads was selected to perform further experiments.

**Fig. 2:**
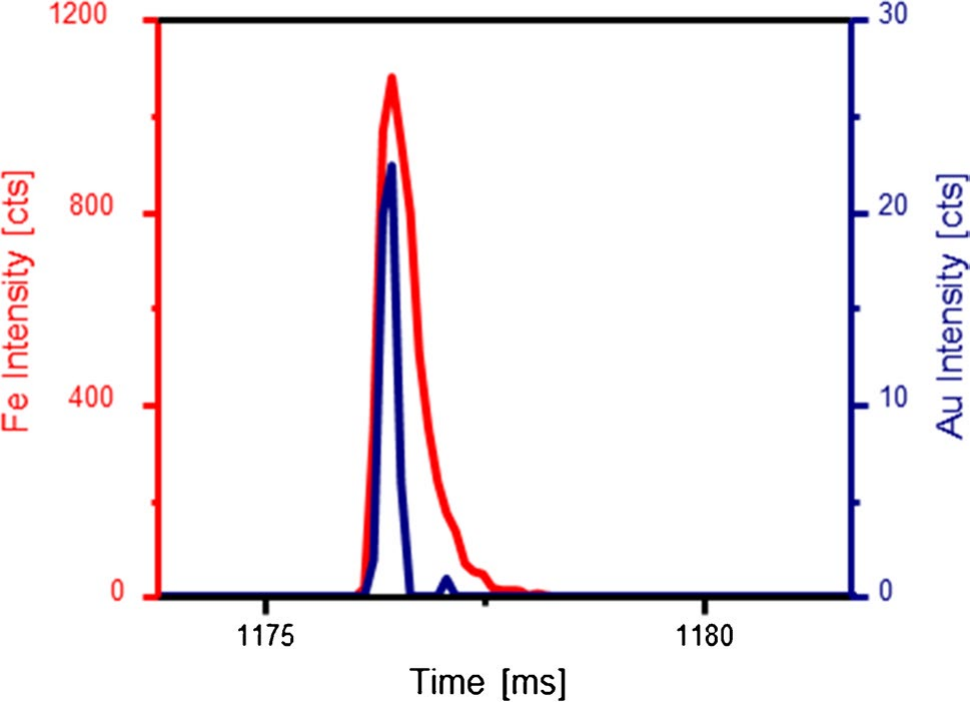
Type of events obtained for the hybrid combination containing the miRNA under study, the Au-probe and the Fe-probe using ICP-TOF–MS

As can be seen, independent from the combination of detection/capture system used, there was a significant contribution of just gold containing events (solid part of the bars) even after thorough cleaning of the assay. These were only detection probes that were not hybridized with the analyte or the capture probe. Figure 2 shows the simultaneous presence of Fe (red trace) and Au (blue) in one of the events obtained applying the developed strategy to 1500 pM of the miRNA in the SP-ICP-ToF–MS revealing the capabilities of this type of instrumentation to discriminate between just Au or Au- and Fe-containing signals.

As mentioned before, only gold-containing events are always present, even after thorough washing of the samples. The possibility of counting only the events that contain Au and Fe at the same time improves the selectivity of this strategy, since only those events produced by the complete sandwich (analyte, reporter and capture probe) are considered and counted, avoiding the counting of unspecifically adsorbed gold nanoparticles. This is of special importance in the case of intracellular miRNA analysis, since the presence of many different miRNA sequences that may contain even one-nucleotide variations is here more expected in comparison to other biological samples like blood.

### Optimization of the concentration of the oligo/NPs ratio for maximum sensitivity

Aiming to increase the sensitivity of the assay, the minimum concentration of the detection oligonucleotide necessary to maintain the recognition capabilities in the detection probe for the analyte was evaluated. Two procedures were conducted: first, the concentration of Au NPs was kept constant while the concentration of oligo was reduced in successive experiments from the initial conditions and the response tested SP-ICP-ToF–MS monitoring events containing both Au and Fe. In a second experiment, the concentration of oligo was kept constant while the number of particles was reduced. In the first case, a linear response with the analyte concentration was obtained when halving the amount of detection probe (Figure S3). Lower concentrations of oligo showed similar blank values but a low linearity of the response against higher analyte concentrations.

**Fig. 3:**
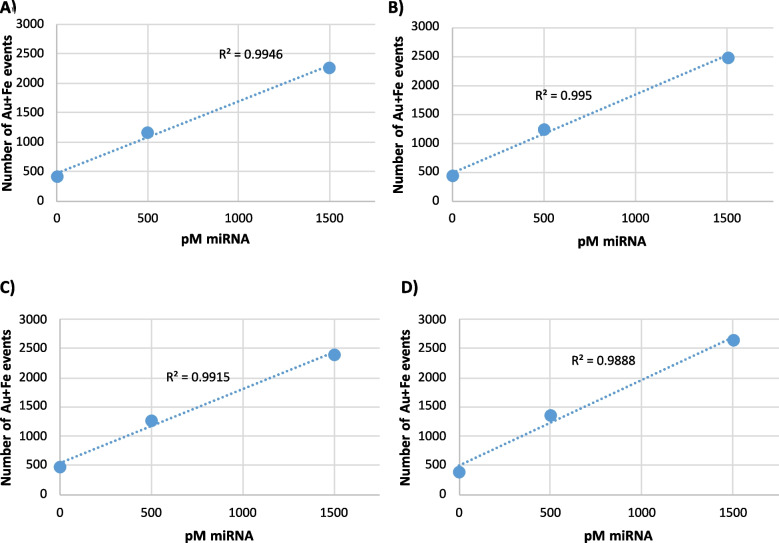
Regression lines obtained for the Au-Fe containing events obtained upon increasing the miRNA concentration (pM) for **A** 1.6 × 10^11^, **B** 1 × 10^11^, **C** 7 × 10^10^, and **D** 3.2 × 10^10^ particles per milliliter

In the case of maintaining a constant concentration of the detection oligo and decreasing the number of particles, the results are shown in Fig. 3. The regression models were compared using ANOVA for model comparison. *P*-values were, in all cases, higher than 0.05. Therefore, there are no significant differences in the linearity of the assays when decreasing the number of particles in the final assay from 1.6 × 10^11^ (Fig. 3A) to 3.2 × 10^10^ (Fig. 3D).

Further dilutions showed a loss of linearity (not shown) and thus the final number of particles was adjusted to conditions used in Fig. 3D and with a mass of oligo of about 400 pmol. It is worth mentioning that every data point of the calibration curves (in Figs. 3 and S3) is obtained in an independent assay. Nevertheless, the blank levels when comparing events containing gold and iron show remarkably similar values and always around 450–500 events. These results point out, on the one hand, the reproducibility of the assay and, in another, the limitation to increase sensitivity affected by the unspecific blank signals that cannot be lowered, even when different cleaning strategies were previously tested [18].

**Fig. 4:**
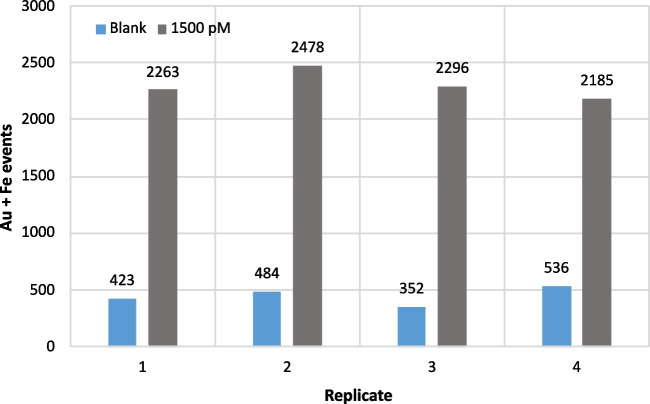
Reproducibility of four independent replicates of the assay. Blue bars (Au events) corresponding to the blanks and grey bars (Au + Fe events) corresponding to independent replicates of 1500 pM

The limit of detection was calculated using these set of conditions and turned out to be about 160 pM of miRNA. Under optimum conditions, a study on the reproducibility of the assay at 1500 pM concentration of miRNA was done obtaining the results of Fig. 4. The reproducibility among replicates is below 10%.

While the reproducibility of our method provides a perfectly acceptable value, the limit of detection is slightly higher in comparison to other published methods. For example, we previously published a similar strategy with a limit of detection of 10.7 pM [18]. However, in the present work, we have slightly modified the assay to use bigger detection nanoparticles and the biotin-streptavidin interaction for the conjugation, instead of a thiol-based interaction. These changes would be sufficient to explain the different limit of detection, but in this case, the matrix is also totally different because the assay is now applied to cell culture lysates, instead of blood serum.

In terms of limits of detection, other SP-ICP-MS-based methods for nucleic acid sequence quantification have reported limits of detection in the low-picomolar or even femtomolar range. However, two aspects should be noted. Firstly, most methods do not address the quantification of naturally present miRNAs in a natural matrix. Instead, they produce spiked samples to study the analytical figures of merit. Moreover, our method addresses the quantification of a sequence with only 22 nucleotides, while other SP-ICP-MS-based methods are applied to the quantification of longer sequences of up to 50 nucleotides [16, 17, 25–29] or even complete genes [30].

Traditional amplification-based protocols, like RT-PCR, also show typical limits of detection in the (low) femtomolar range [31]. Some novel developments can even decrease the detection limit down to the attomolar level [32]. However, standardization and primer design are major challenges in amplification-based strategies that can be overcome with amplification-free methods.

In general, it must also be noted that comparison of limits of detection in this case is complicated. Most published methods report their limits of detection in units of concentration. However, very few provide information about the amount of sample that is used for the experiment, thus hindering the direct comparison of the limits of detection with regard to the total analyte mass. Although comparing limits of detection within the same matrix as concentration is perfectly valid, doing the same with different matrices, as we are trying to do in this discussion, should rather be done in terms of mass of analyte, since the dilution volume is arbitrary and depending on the type of sample. Our limit of detection, as mass of analyte, is 16 fmol of analyte. Since we use a preconcentration step using the magnetic microbeads, this limit of detection is not influenced by the dilution volume of the sample. Instead, the concentration limit of detection can be decreased by increasing the amount of sample used for the assay.

The blank signals, also displayed in Fig. 4, emphasize the challenge of washing out the unreacted particles, but, on the other hand, shows the reproducibility also in the case of the blanks, which allows to subtract the blank signals from the sample signals when the assay is applied to real samples. Even in that case, decreasing the number of blank signals, probably due to the unspecific adsorption of gold nanoparticles on the surface of the magnetic microbeads, would be desirable in order to decrease the limits of detection. However, these could not be further decreased even after a larger number of washing steps.

### Application of the methodology to the analysis of miR-16-5p to cell cultures

As previously stated, miR-16-5p shows important roles in the development of diverse malignancies including neuroblastoma, osteosarcoma, hepatocellular carcinoma, etc. [3]. Therefore, its role as biomarker of these malignancies is well stablished [1, 2]. Previous studies indicated that melanoma cell lines, specifically the model A375, is among the top 20 cell models expressing the sought miRNA and, therefore, this cell line was selected to initiate the studies in real samples [33]. Two different sample preparation strategies were used, considering the potential presence of the analyte within the cell cytosol: (1) the cell lysis and direct analysis of miR-16-5p in the lysate and (2) isolation of total RNA using an established protocol and analysis of the sought sequence with the proposed strategy.

**Fig. 5:**
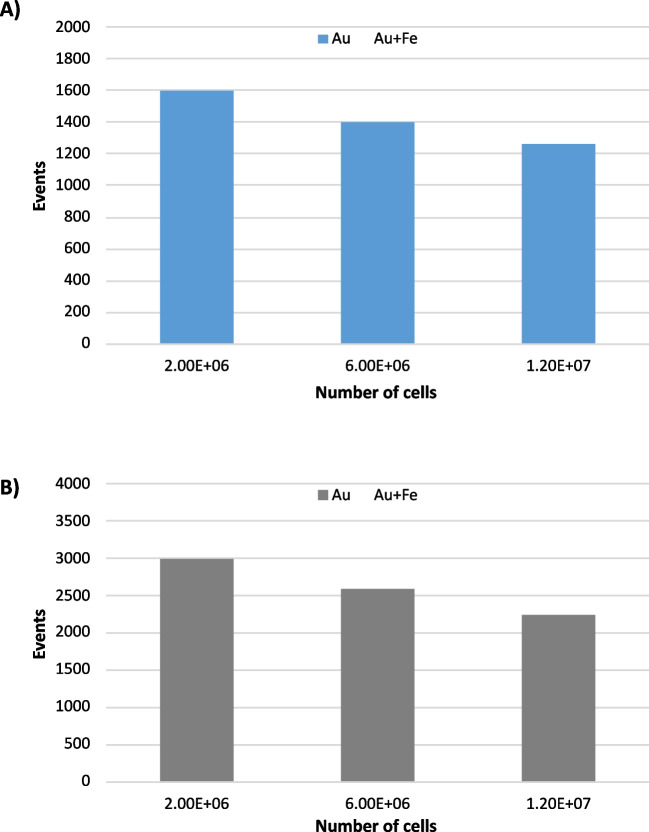
Results obtained from cell extracts taking different number of cells **A** in the crude cell extract and **B** in the pooled extracted RNA. Solid bars (Au events) correspond to the blank and dotted bars (Au + Fe events) corresponding to the increasing amounts of the miRNA. Error bars show the standard deviation for 3 replicates

For the first protocol, an ultrafiltration was performed after cell lysis so that the residual membranes and organelles were eliminated. This filtration step was mandatory since the direct assay on the cell lysate caused the aggregation of the nanoparticles (see Fig. S4). The obtained results are shown in Fig. 5A for different numbers of lysed cells. Although the number of gold events was relatively high even with the lowest number of cells, the number of events containing both Au and Fe were below those observed for the blank of the assay (see Fig. 3). As previously observed during method development, this was likely due to the unspecific adsorption of the Au-probes to different molecules within the cytosol, reducing the capabilities of the capture probe to recover the miR-16-5p molecules. When increasing the number of cells, an increase of the number of events containing Au + Fe revealed the capabilities of the assay to reflect the increase in the miR-15-5p concentration. However, these levels were still below the calibration blank.

Thus, an additional sample preparation step was conducted in order to selectively isolate RNA from the cells using the TRIZol protocol and then conduct the capturing from this extract. The purity of extracted RNA (obtained spectrophotometrically) was obtained for 2 × 10^6^, 6 × 10^6^, and 12 × 10^6^ cells with a mean value (ratio 260/280 nm) of 1.93 (ratios of about ~ 2.0 are considered as pure RNA). The results obtained for this assay are shown in Fig. 5B. The number of Au events fitted within the calibration range (see Fig. 1A). In addition, the Au + Fe event number was always above the calibration blank and statistically different between the three cell number conditions (*p* < 0.05 in a Student *t* test). These values permitted the quantification of miR-16-5p even in the 2 × 10^6^ cells lysate which turned out to be about 200 pM (close to the calculated limit of detection of the method). For the samples with a higher cell number, the results obtained corresponded to 427 pM (6 × 10^6^ cells) and 636 pM (12 × 10^6^ cells). By triplicating the number of cells (from 2 × 10^6^ to 6 × 10^6^), the increase of the obtained concentration corresponded to about 70% of what was expected. Further increase in the cell number concentrations (from 6 × 10^6^ to 12 × 10^6^) yielded on an increase of about 75% with respect to what was expected. This could be also ascribed to the overall RNA extraction yields obtained for each experiment, which could be highly dependent on the number of cells. Therefore, the extraction of the miR-16-5p could be also affected by the inaccuracy of such procedure. In any case, the obtained results show adequate suitability for miR-16-5p quantification in the cell extracts to be used for comparative purpose among cell types.

As previously mentioned, the strategy that has been developed has some limitations, specially in sensitivity, and mainly due to the high contribution of the blanks due to the unspecific adsorption of the gold nanoparticles onto surfaces. To overcome this issue, new coatings of the reporter gold nanoparticles should be investigated that minimize secondary and unspecific interactions. Nevertheless, the method has been useful to quantify the miR-16-5p naturally present in real cancer cells without any fortification. In addition, the methodology that has been developed here can be tuned to detect any other target miRNA by only substituting the capture and reporter probes, and it could even go multiplex by additionally using reporter nanoparticles with different compositions or sizes. Finally, as demonstrated in our previous publication [18], a similar strategy has been validated in blood serum. Although blood serum is a more complex matrix, the variability of the miRNAs present is expected to be much lower than those present in the cell cytosol. Therefore, the additional selectivity filter provided by the ICP-ToF–MS and the dual particle detection is crucial in the determination of miRNAs in cell extracts.

## Conclusions

The double hybrid sandwich assay using 40 nm Au nanoparticles as detection probe and magnetic Fe microparticles as capture probe, both coated with streptavidin, provided good selectivity towards miR-16-5p. Eliminating the need for nucleic acid sequence amplification, our approach streamlined the analysis process while reducing potential sources of error that are common in conventional techniques. The optimization of the reagents permitted to maximize the stoichiometry aiming to improve the method sensitivity. In addition, the combination of the assay with the use of SP-ICP-ToF–MS as detector permits a double monitoring of Fe and Au, facilitating the confirmation of the hybrid formation and the discrimination of the contribution of the blanks due to unspecific adsorption of some of the probes. As such, restricting the detection to the condition that events must contain Fe and Au adds selectivity and sensitivity to the proposed assay. The final approach has been successfully tested to quantify the sought sequence in cell lysates of melanoma showing the possibility of absolute quantification after RNA extraction.

## Supplementary Information

Below is the link to the electronic supplementary material.

ESM 1 (DOCX 218 KB)

## Data Availability

Research data generated for this publication will be available on request.
