# Supplementary material for: Quantitative determination of intracellular miRNA content using dual gold and iron nanoreporters and single particle ICP-ToF–MS
Source: Mikrochim Acta. 2025 Jun 2;192(6):390. doi: 10.1007/s00604-025-07236-4 (PMC12129863; doi:10.1007/s00604-025-07236-4)
Supplement: Supplementary file 1 — (DOCX 218 KB) [file 604_2025_7236_MOESM1_ESM.docx]

**SUPPLEMENTARY MATERIAL**

**Quantitative determination of intracellular mirna content using dual gold and iron nanoreporters and single particle ICP-ToF–MS**

Sara González Morales,^1^ Elena Añón Álvarez,^3^ David Clases,^2^ Mario Corte-Rodriguez^1,3^* and Maria Montes-Bayón^1,3^*.

^1^Department of Physical and Analytical Chemistry, Faculty of Chemistry, University of Oviedo, Julián Clavería 8, 33006 Oviedo, Spain.

^2^Department of Chemistry. University of Graz.

^3^Health Research Institute of the Principality of Asturias (ISPA), Av. Hospital Universitario s/n, 33011 Oviedo, Spain.

[*montesmaria@uniovi.es](mailto:*montesmaria@uniovi.es); [*cortemario@uniovi.es](mailto:*cortemario@uniovi.es)

**
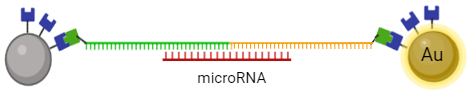
**

Biotinylated oligonucleotides

**40 nm** Streptavidin coated Au NP

Sera-Mag™ SpeedBead Streptavidin (**1 µm**)

**Figure S1.** Schematic diagram of the developed assay

**A)**


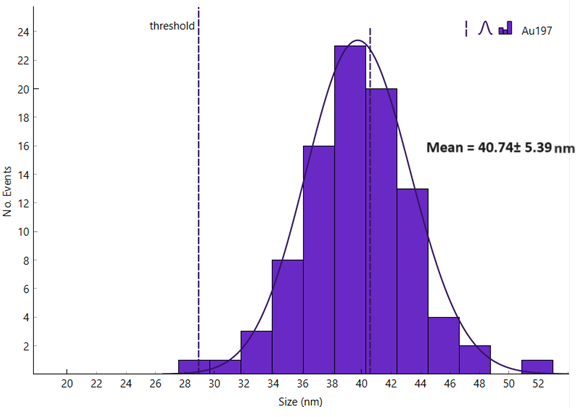


**B)**

**
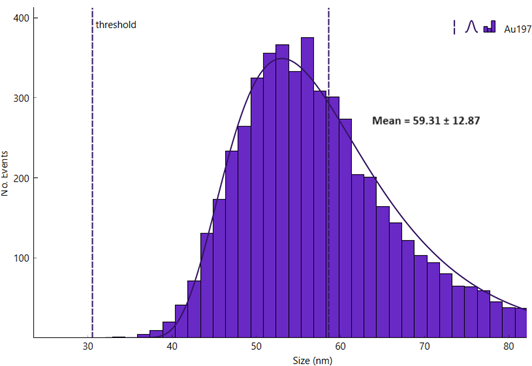
**

**Figure S2.** Histograms corresponding to the analysis of the two Au NPs used in the assay by SP-ICP-ToF-MS. A) 40 nm and B) 60 nm.


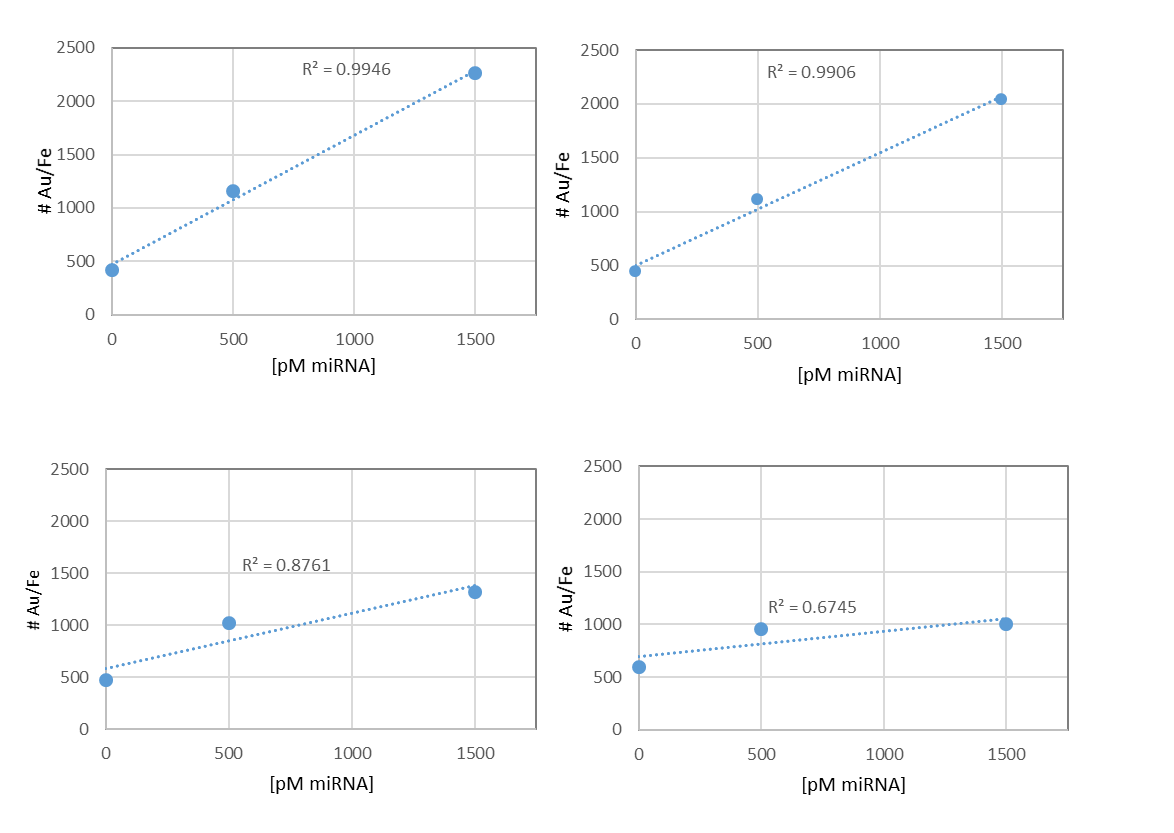


**Figure S3.** Response of the assay to increasing concentrations of the analyte while decreasing the concentration of the detection probe. A) 858 pmol, B) 424, C) 255 and D) 170 pmol.


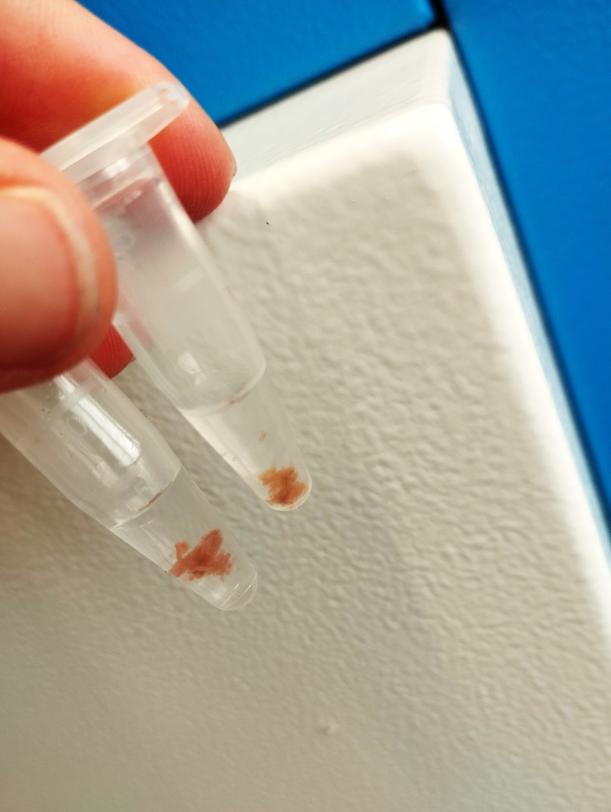


**Figure S4.** Aggregates observed after conducting the assay directly on the cell lysate before ultrafiltration.
